# Meleney’s Gangrene of the Abdomen Managed With Serial Debridement and Negative Pressure Wound Therapy: A Case Report

**DOI:** 10.7759/cureus.68440

**Published:** 2024-09-02

**Authors:** Aditya Sharma, Vivek K Katiyar, Satyendra K Tiwary, Puneet Kumar, Ajay K Khanna

**Affiliations:** 1 Department of General Surgery, Institute of Medical Sciences, Banaras Hindu University, Varanasi, IND

**Keywords:** high mortality, skin grafting, negative pressure wound therapy (npwt), serial debridement, meleney's gangrene

## Abstract

Meleney's gangrene (necrotizing fasciitis (NF)), also known as progressive bacterial synergistic gangrene, is a potentially fatal subcutaneous tissue infection with abdominal wall necrosis that progresses rapidly and systematically. It has been observed to exhibit the cultural characteristics of a symbiotic organism. Due to its rarity and high mortality rate, this infection needs to be diagnosed promptly and treated aggressively with antibiotics and rigorous debridement. There are several approaches to management, which include intravenous antibiotics, aggressive debridement, and dressings, along with the application of negative pressure wound therapy (NPWT). Herein, we report the case of a 45-year-old male patient with type 2 diabetes mellitus who presented to our facility after being bitten by an insect and exhibiting symptoms of Meleney's gangrene of the abdomen.

## Introduction

Necrotizing fasciitis (NF) of the abdominal wall is an infectious condition that can be fatal and needs to be treated promptly with medical care and surgery [1]. This disease progresses quickly and develops necroses that spread to the fascia and subcutaneous tissue. One of the disease's hallmarks is its early potential to cause systemic toxicity [2]. Sepsis, acute respiratory distress syndrome (ARDS), disseminated intravascular coagulation (DIC), septic shock, acute renal failure, liver failure, and multiple organ failures are common causes of death [1,2]. The microbiological effectors for Meleney's gangrene were initially characterized by Dr. Meleney and Dr. Brewer in 1926, and Dr. Meleney further classified them in 1931 [2]. It is a potentially fatal infection of the skin and subcutaneous tissue of the abdomen that advances rapidly. It is a devastating subcutaneous tissue infection that is uncommon, fast-progressing, and difficult to diagnose because of its nonspecific initial symptoms [1]. The severity of this condition is thought to be caused by the synergistic effects of several bacteria [1,2]. The synergistic infection caused by *Staphylococcus aureus* and Streptococcus species results in Meleney's gangrene [2,3]. The most common cases occur after surgery in the lower abdomen or trauma, and it is more prevalent in diabetics and immunocompromised individuals. Since several bacteria can be destructive, it is known as synergistic gangrene [2]. The majority of documented Meleney's gangrene cases involved wound infections that occurred after surgery, and their occurrence following an insect bite is a rare case scenario that we are reporting [2,3].

## Case presentation

A 45-year-old male presented to the surgical emergency with a 10-day history of swelling and a wound over the lower abdominal wall following an insect bite. He was working in an open space when he was stung in the left lower quadrant of his abdomen by a hoverfly that initially looked harmless. At first, nothing was evident, so even though he felt the bite, he did not take it seriously. The patient, a known case of type 2 diabetes mellitus for the past two years, was taking oral hypoglycemic agents (OHAs). Using his nail, he broke open a little blister at the sting location the following day. He took a painkiller after the lesion gradually started to pain and spread, without seeking any medical consultation. By the sixth day, he developed a fever associated with pain and redness in the affected area, which gradually progressed to the whole abdomen within 10 days. Thereafter, he visited a local practitioner, who referred him to our healthcare center. On examination, the patient was febrile, exhibited tachycardia, and showed signs of dehydration. Upon local examination, there was generalized cellulitis involving the entire abdomen, along with a few regions of ulceration. On palpation, there was a local rise in temperature, tenderness was present, and foul-smelling seropurulent discharge was noted from the wound edges. The perianal region, scrotum, and external genitalia were all found to be normal. The diagnosis of NF was established clinically. A polymicrobial infection (gram-positive *Streptococcus pyogenes*; gram-negative bacteria: *Pseudomonas aeruginosa* and *Klebsiella pneumoniae*) was identified using wound culture and sensitivity. Subcutaneous air pockets were seen on an abdominal ultrasound, which ruled out intraperitoneal involvement. After proper resuscitation and administering IV sedation, emergency debridement was performed on the patient after obtaining informed consent. The patient received IV antibiotics and appropriate glycemic control treatment in the post-operative ward following the endocrinology consultation. Laboratory blood parameters during the admission are shown in Table 1.

**Table 1:** Laboratory blood parameters of the patient at the time of admission.

Parameter	Patient Value	Reference Range
Total Leucocyte Count (TLC)	26,000/µl	4,000-10,000/µl
Blood Urea	48 mg/dl	17-50 mg/dl
Serum Creatinine	1.1 mg/dl	0.8-1.3 mg/dl
Random Blood Sugar (RBS)	287 mg/dl	70-140 mg/dl

**Figure 1:**
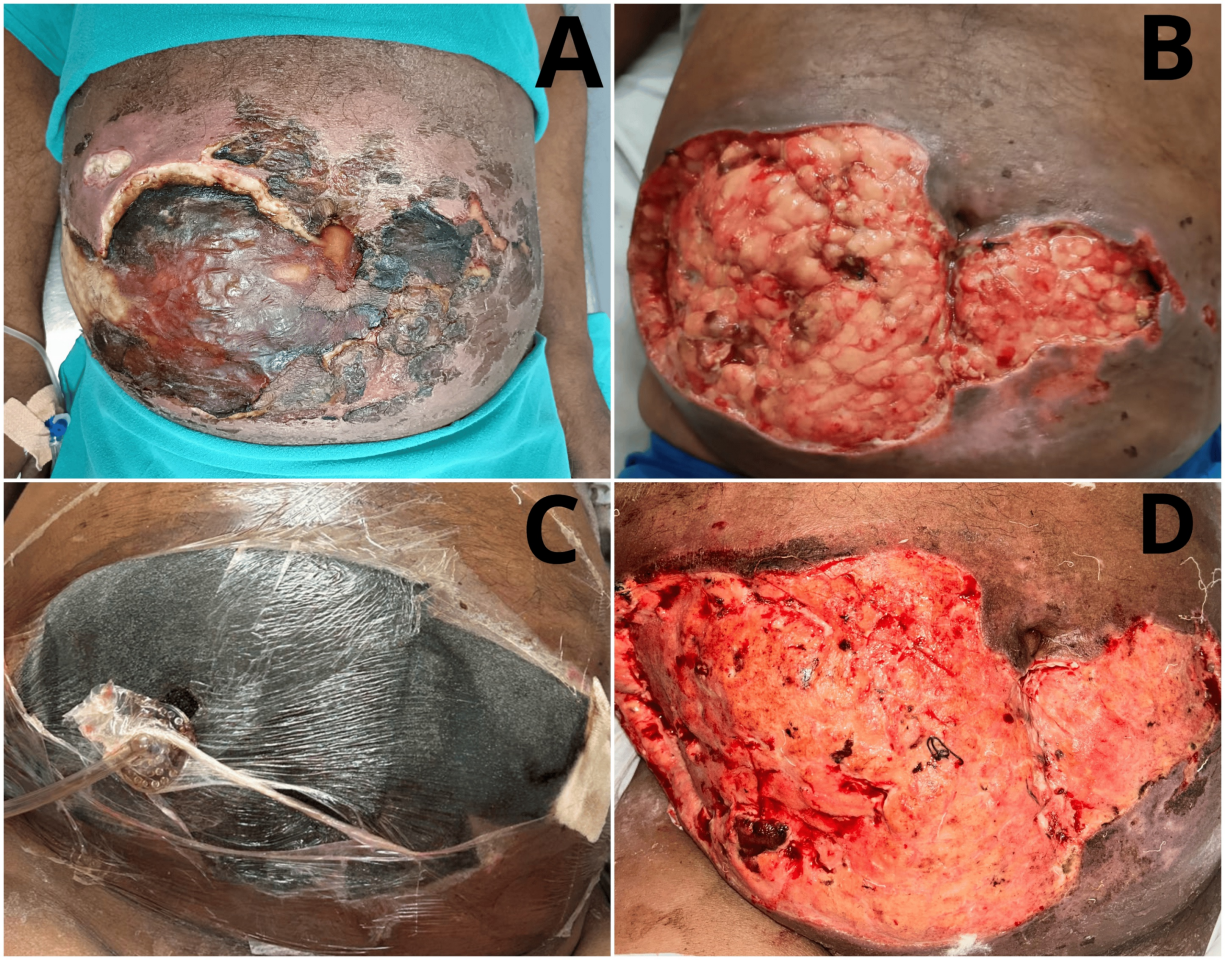
Clinical images: (A) pre-debridement; (B) post-debridement; (C) after application of NPWT; and (D) raw granulation area after removal of NPWT. NPWT: Negative pressure wound therapy.

The next day, the application of NPWT was initiated. A device providing a negative pressure of 125 mm Hg was connected to the tubing that emerged from the sealing drape. The pump ran on a 7-minute cycle, turning on for five minutes and shutting off for two minutes. Over the following few days, the patient's renal parameters and total count returned to normal. With daily debridement and sterile dressing application, along with the use of NPWT, the condition of the wound improved. NPWT was removed on day 7 of the admission, following which regular dressing of the raw area was performed. On day 12, the patient was discharged with a healthy granulating wound and normal blood counts. He was referred to plastic and reconstructive surgery for skin grafting and further follow-up. The patient was planned for split thickness skin grafting (STSG) from the thigh region to cover the raw area produced as a result of serial debridement; the graft took well, and the raw area healed completely within 2-3 weeks following the procedure. The patient did well in his follow-up visits to the concerned department. The pre-debridement, post-debridement, and after application of NPWT and its removal clinical images are shown in Figure 1.

## Discussion

NF is a soft tissue infection often brought on by mixed infections involving gram-negative bacteria, coliforms, and anaerobes [2]. It is characterized by widespread inflammation of the skin, deep fascia, and soft tissues, as well as significant tissue destruction. Important predisposing factors include diabetes, drug addiction, alcoholism, obesity, malnutrition, malignancies, immunodeficiency, and other chronic medical disorders. It was first described by Hippocrates in the early fifth century BC [2,3]. Meleney illustrated this synergy by using the development of the usual gangrenous lesion upon injection of both species into the skin of dogs.

Meleney's ulcer is mainly caused by a subcutaneous tissue infection that results in subcutaneous necrosis and small-vessel thrombosis, urgently necessitating surgery. Improved prognosis critically depends on early diagnosis, typically determined by clinical methods [4,5]. It is important to diagnose Meleney's gangrene as soon as possible and treat it using aggressive surgical debridement and broad-spectrum antibiotics. Differential diagnosis may include other soft tissue infections such as Fournier gangrene and Ludwig's angina, as well as other pyogenic superficial skin infections. Early diagnosis of the illness is challenging, and there is a great deal of misunderstanding during this time. A delayed diagnosis of Meleney's gangrene may be associated with a higher likelihood of death. Serial debridement and regular dressing may necessitate an extended hospital stay. Moreover, in some cases, skin grafts may be required or necessary for the healing of wounds with larger raw areas.

The first course of treatment for NF is aggressive surgical debridement, followed by targeted antibiotic therapy and the application of NPWT to hasten the wound healing process and promote early recovery [6]. NPWT stimulates modulation of numerous local and circulating cytokines and growth factor expressions to promote an anti-inflammatory profile. This is most likely achieved by the downregulation of TNF-α, upregulation of vascular endothelial growth factor (VEGF), TGF-β, and fibronectin. NPWT, has been utilized for the past 20 years to treat various non-healing ulcers, including pressure sores. Since 1995, this technique, which uses vacuum-assisted closure, or VAC, has been applied in clinical settings. Through the use of polyvinyl alcohol foam or polyurethane reticulated open cell foam (ROCF), this technique provides localized negative pressure to the wound bed. It facilitates reduced local edema, enhanced wound edge vascularity, elimination of inhibitory chemicals, stimulation of granulation tissue formation, provision of a moist wound healing environment, and promotion of wound contraction, all facilitated by negative pressure up to 125 mm of Hg [6].

It is imperative to remove any necrotic fascia, non-viable skin, and subcutaneous tissue [7]. The necrosis spreads quickly; thus, this must be done as often as required. Debridement is usually necessary well into the healthy area since the affected tissue typically extends beyond the clearly visible borders. It is advised to perform regular wound checks and postoperative dressing changes [8,9]. The umbrella term for Meleney's gangrene is 'necrotizing fasciitis.' With a 34% fatality rate, NF is a potentially fatal illness [4]. A rare type of NF known as Meleney's gangrene is frequently observed in immunocompromised conditions such as post-surgery, diabetes mellitus, old age, HIV, etc. It begins as a superficial skin infection or ulcer that rapidly spreads to the subcutaneous tissue, followed by widespread subcutaneous plane spreading, microscopic vascular thrombosis that results in necrosis, and ultimately gangrene [4,5,10].

The truncal area is usually where Meleney's gangrene is seen. When injected separately, neither organism was able to produce a lesion of this kind. After an insect bite, trauma, or surgery, a tiny, superficial ulcer is typically the first sign of Meleney's ulcer [5,10]. It could also result from lymph nodes that are infected [6,10]. Typically, after debridement, the raw area is covered with a skin graft taken from the thigh, and dressing is continued until healthy granulation tissue develops. However, wound repair using the abdominoplasty procedure was envisioned because the soft tissue loss was irreversible. For cosmetic reasons, abdominoplasty is a commonly performed operation. Few cases of therapeutic abdominoplasty have been documented in international literature [10].

Surgeons need to be aware of NF since it is a clinically challenging condition. Radical surgical debridement and early diagnosis are necessary. If NF is clinically suspected, surgery should be considered and should not be postponed due to diagnostic delays in order to reduce mortality.

## Conclusions

Unfortunately, many of Meleney's gangrene symptoms are initially neglected in tropical regions, which needlessly raises the mortality rate. Meleney's gangrene may be considered a differential diagnosis in the postoperative patient with sepsis, wound dehiscence, and drainage from the surgical site, as well as following trauma or an insect bite. The key components of treatment include aggressive serial debridement combined with antibiotic coverage and the use of NPWT (optional), to promote early healing of the wound. It is important to inform healthcare professionals in peripheral areas about this condition so that it can be promptly diagnosed and optimally managed. The purpose of presenting this case is to highlight how Meleney's gangrene, a rare and clinically significant condition, can be treated more successfully with early intervention and aggressive debridement.

## References

[1] Dworkin MS, Westercamp MD, Park L, McIntyre A (2009). The epidemiology of necrotizing fasciitis including factors associated with death and amputation. Epidemiol Infect.

[2] Kapi E, Dogan ZDA, Seyhan T (2018). Unusual cases of necrotizing fasciitis: a clinical experience from Turkey. Eur J Plast Surg.

[3] Freischlag JA, Ajalat G, Busuttil RW (1985). Treatment of necrotizing soft tissue infections. The need for a new approach. Am J Surg.

[4] Huang KF, Hung MH, Lin YS, Lu CL, Liu C, Chen CC, Lee YH (2011). Independent predictors of mortality for necrotizing fasciitis: a retrospective analysis in a single institution. J Trauma.

[5] Khanna AK, Tiwary SK, Kumar P, Khanna R, Khanna A (2009). A case series describing 118 patients with lower limb necrotizing fasciitis. Int J Low Extrem Wounds.

[6] Childers BJ, Potyondy LD, Nachreiner R (2002). Necrotizing fasciitis: a fourteen-year retrospective study of 163 consecutive patients. Am Surg.

[7] Bair MJ, Chi H, Wang WS, Hsiao YC, Chiang RA, Chang KY (2009). Necrotizing fasciitis in southeast Taiwan: clinical features, microbiology, and prognosis. Int J Infect Dis.

[8] Hsiao CT, Weng HH, Yuan YD, Chen CT, Chen IC (2008). Predictors of mortality in patients with necrotizing fasciitis. Am J Emerg Med.

[9] Golger A, Ching S, Goldsmith CH, Pennie RA, Bain JR (2007). Mortality in patients with necrotizing fasciitis. Plast Reconstr Surg.

[10] Ahmed S, Maharjan N, Hirachan N (2024). Meleney's gangrene managed with a single extensive debridement and resultant defect closure with abdominoplasty technique - a case report. Ann Med Surg (Lond).

